# Major hepatectomy for perihilar cholangiocarcinoma in elderly patients: is it reasonable?

**DOI:** 10.1007/s13304-021-01111-6

**Published:** 2021-06-17

**Authors:** L. Ripamonti, R. De Carlis, A. Lauterio, I. Mangoni, S. Frassoni, V. Bagnardi, L. Centonze, C. Poli, V. Buscemi, F. Ferla, L. De Carlis

**Affiliations:** 1 Department of General Surgery and Transplantation, ASST Grande Ospedale Metropolitano Niguarda, Milan, Italy; 2 Department of Medicine and Surgery, University of Milano-Bicocca, Milan, Italy; 3 Department of Statistics and Quantitative Methods, University of Milan-Bicocca, Milan, Italy

**Keywords:** Perihilar, Cholangiocarcinoma, Elderly, Hepatectomy, Liver, Klatskin

## Abstract

**Introduction:**

We sought to evaluate the effect of age on postoperative outcomes among patients undergoing major liver surgery for perihilar cholangiocarcinoma (PHCC).

**Methods:**

77 patients were included. Patients were categorized into two groups: the “< 70-year-olds” group (*n* = 54) and the “≥ 70-year-olds” group (*n* = 23).

**Results:**

Median LOS was 19 both for < 70-year-old group and ≥ 70-year-old group (*P* = 0.72). No differences in terms of severe complication were detected (44.4% Clavien–Dindo 3–4–5 in < 70-year-old group vs 47.8% in ≥ 70-year-old group, *P* = 0.60). Within 90 postoperative days, 11 patients died, 6 in < 70-year-old group (11.3%) and 5 in ≥ 70-year-old group (21.7%), *P* = 0.29. The median follow‐up was 20 months. The death rate was 72.2% and 78.3% among patients < 70 years old and ≥ 70 years old. The OS at 2 and 5 years was significantly higher among the < 70 years old (57.0% and 27.7%) compared to the ≥ 70 years old (27.1% and 13.6%), *P* = 0.043. Adjusting for hypertension and Charlson comorbidity index in a multivariate analysis, the HR for age was 1.93 (95% CI 0.84–4.44), *P* = 0.12. Relapse occurred in 43 (81.1%) patients in the < 70-year-old group and in 19 (82.6%) patients in the ≥ 70-year-old group. DFS at 12, 24, and 36 months was, respectively, 59.6, 34.2, and 23.2 for the < 70 -year-old group and 32.5, 20.3, and 13.5 for the ≥ 70-year-old group (*P* = 0.26). Adjusting for hypertension and Charlson comorbidity index in a Cox model, the HR for age was 1.52 (95% CI 0.67–3.46), with *P* = 0.32.

**Conclusions:**

≥ 70-year-old patients with PHCC can still be eligible for major liver resection with acceptable complication rates and should not be precluded a priori from a radical treatment.

## Introduction

Perihilar cholangiocarcinoma (PHCC), or Klatskin tumor, is an advanced tumor at or near the confluence of the right and left hepatic duct. With approximately 5,000 new cases diagnosed annually in the United States, PHCC represents less than 2% of all malignancies, yet accounts for over 60% of all cholangiocarcinomas. Surgical resection is the only way to cure this disease, as chemotherapy with or without radiation is less effective [1–3]. Due to the difficulty, risks, and complexity of resection, hepatectomy has rarely been offered to elderly patients with PHCC. Older patients often have a higher incidence of medical comorbidities, worse performance status, as well as decreased functional reserve that may place them at particular risk for worse postoperative outcomes [4]. The incidence of Klatskin tumors increases with age, with a peak between 60 and 80 years [5]. Improvements in living conditions and progress in medical and surgical management have resulted in aging of the population. According to the Italian National Institute for Statistics (Istituto Nazionale di Statistica, ISTAT), people aged 70 and older in Italy in 2017 represent 17% of the inhabitants with a life expectancy of 83 years (instead 13% and 80 years in 2003) [6, 7]. In consideration of this improvement in life expectancy, it is of great importance to treat elderly people properly by attempting to offer them radical surgery in accordance with their comorbidities and functional status. Different studies treating different liver tumors have proven the safety and feasibility of liver resection for elderly patients with an acceptably low complication rate and adequate oncologic outcomes [8–10]. In this study, we sought to evaluate the effect of age on postoperative outcomes among patients undergoing liver surgery for PHCC. In particular, we aimed to compare perioperative outcomes including postoperative complications, 90-day mortality, and length of stay (LOS), as well as oncological outcomes like disease-free survival (DFS) and overall survival (OS) between elderly patients (≥ 70 years old) and younger (< 70 years old) undergoing liver major resection for PHCC.

## Methods

**Fig. 1:**
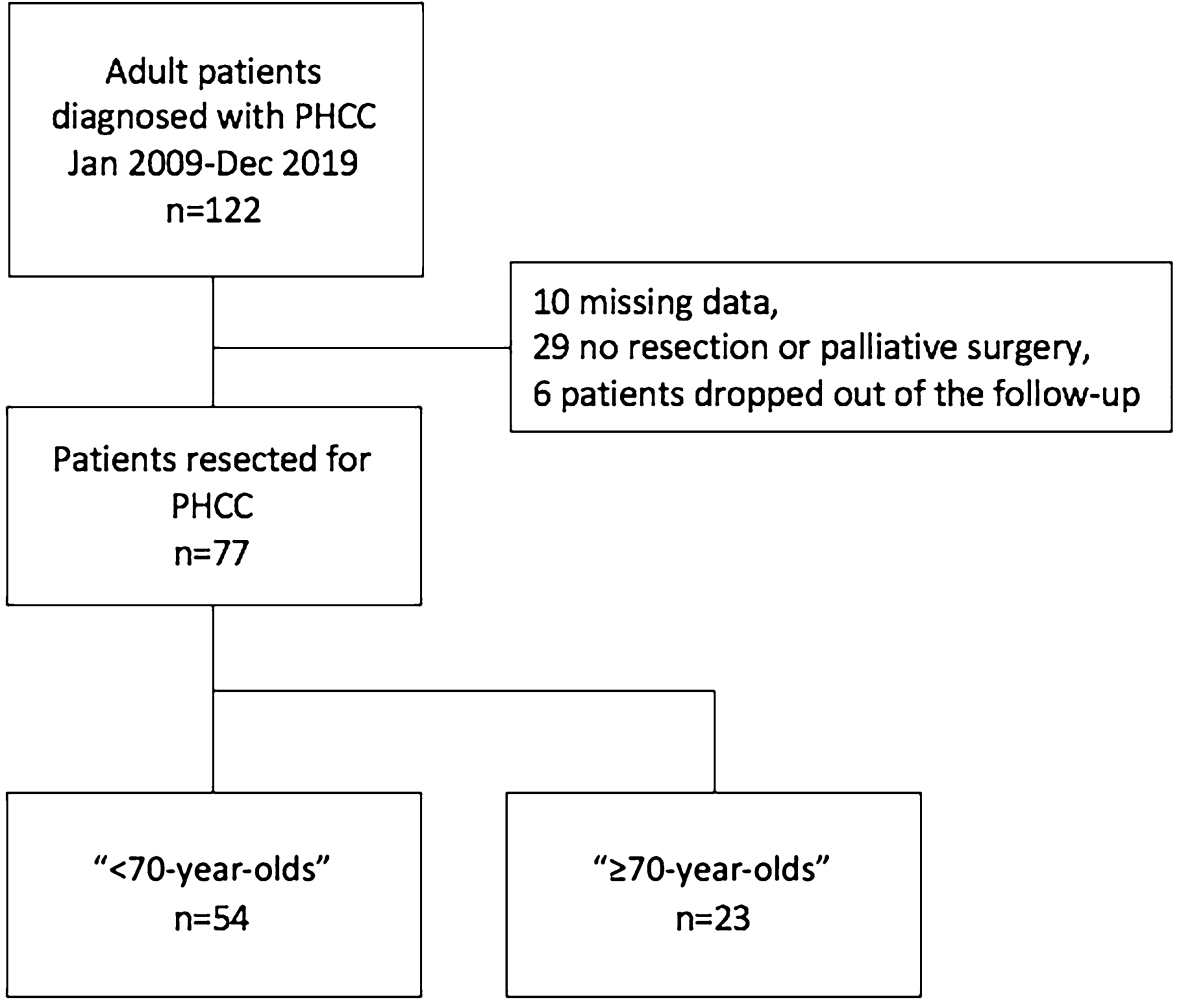
Patient selection

The study evaluated consecutive 122 adult (≥ 18 years) patients diagnosed with PHCC and recruited between January 2009 and December 2019 at our Institution. The pathology of PHCC was diagnosed according to the World Health Organization and AJCC criteria [11, 12]. Intrahepatic and distal cholangiocarcinoma, gallbladder carcinoma, hepatocellular carcinoma, and all secondary liver malignancies were excluded. Data were collected retrospectively and anonymized prior to the analysis. Ten patients were excluded due to missing data and 29 patients were excluded, because only surgical exploration without resection or palliative surgery was performed and 6 patients dropped out of the follow-up. Overall, 77 patients resected for PHCC were included in the study. Patients were categorized into two groups according to the different age: the “< 70-year-olds” group (age < 70, *n* = 54) and the “ ≥ 70-year-olds” group (age ≥ 70, *n* = 23) (Fig. 1). For each group, baseline characteristics, post‐operative results, and survival were evaluated.

All patients signed informed consent that data and follow-up will be collected anonymously and is potentially used for scientific analysis. This study complied with the standards of the Declaration of Helsinki and current ethical guidelines, and no ethical approval was necessary owing to the retrospective, observational, and anonymous nature of this study.

### Preoperative management and follow-up

Preoperative imaging examinations, including contrast-enhanced computer tomography (CT) and/or magnetic resonance imaging (MRI), were performed routinely and reviewed for this study by an expert radiologist. Serum biochemical was used to assess liver function. Considering the potential risk of postoperative complications and hilar sclerosis associated with preoperative biliary drainage, we considered drainage for patients who presented with preoperative cholangitis, experienced a significant delay in surgery for various reasons, or for whom we planned an extensive resection (e.g., trisectionectomy). Otherwise, we tolerated a moderate degree of jaundice and duct dilatation, especially in the case of left hepatectomies. We usually drain only the future liver remnant with percutaneous or, less frequently, endoscopic biliary drainage. Preoperative portal vein embolization (PVE) in our series was considered if a trisectionectomy was planned (with an approximately cut-off of future liver remnant (FLR) of < 25%).

### Surgery

Every hepatectomy had a laparotomic approach. The type of hepatectomy was determined by the location of the primary tumor basing on the Bismuth–Corlette classification. Caudate lobe resection was performed in every case. Lymphadenectomy was always a routine part of the resection procedure. When distant lymph-node metastasis (M1 disease) was found at laparotomy, resection was abandoned in principle.

The parenchymal transection was performed using Cavitron Ultrasonic Surgical Aspirator (CUSA), under both hepatic artery and portal vein clamping for 15 or 20 min (according to surgeon’s preference) at 5-min intervals. Bilioenteric continuity was reestablished by Roux-en-Y cholangiojejunostomy.

### Pathology

Clear resection margin (R0) was defined as the distance between the nontumorous tissue and cancer cell > 1 mm. R1 resection referred to resection margin touching inked tumor on the hepatic section line. The resection margins of the bile duct were investigated by frozen section. R2 resection means gross residual disease on the hepatic section line.

### Outcomes

The primary outcomes were to evaluate the differences between age classes in terms of surgical outcomes (rate of complications, LOS, and 90‐day mortality). Post‐operative complications were recorded and considered as any deviation from the normal recovery requiring pharmacological or interventional treatments. The severity of complication was assessed by the Clavien–Dindo (CD) classification [13] and comprehensive complication index (CCI) [14].

The secondary outcomes were overall survival and disease-free survival among the two groups.

### Statistical analysis

Continuous data are reported as median and ranges. Categorical data are reported as counts and percentages.

Wilcoxon’s signed-rank test for continuous variables, Fisher’s exact test for binary variables, and Cochran–Armitage’s trend test for ordinal variables were used to compare the distribution of the evaluated characteristics between patients under and over 70 years.

To evaluate the effect of age on postoperative outcomes, different univariate models were performed: linear regression model for length of hospital stay and CCI, ordinal logistic model for Clavien–Dindo (3 ordinal levels: 0, 1–2, 3–4–5), and binary logistic model for 90‐day mortality. The overall survival (OS) and disease-free survival (DFS) functions were estimated using the Kaplan–Meier method. The log-rank test was used to assess differences between the two age groups.

Multivariate models (linear, ordinal logistic, binary logistic, and cox proportional hazard) were then performed, to adjust the effect of age at surgery for the baseline characteristics that significantly differed between the two age groups (*P* < 0.05) at univariate analysis.

All analyses were performed with the statistical software SAS 9.4 (SAS Institute, Cary, NC).

## Results

**Fig. 2:**
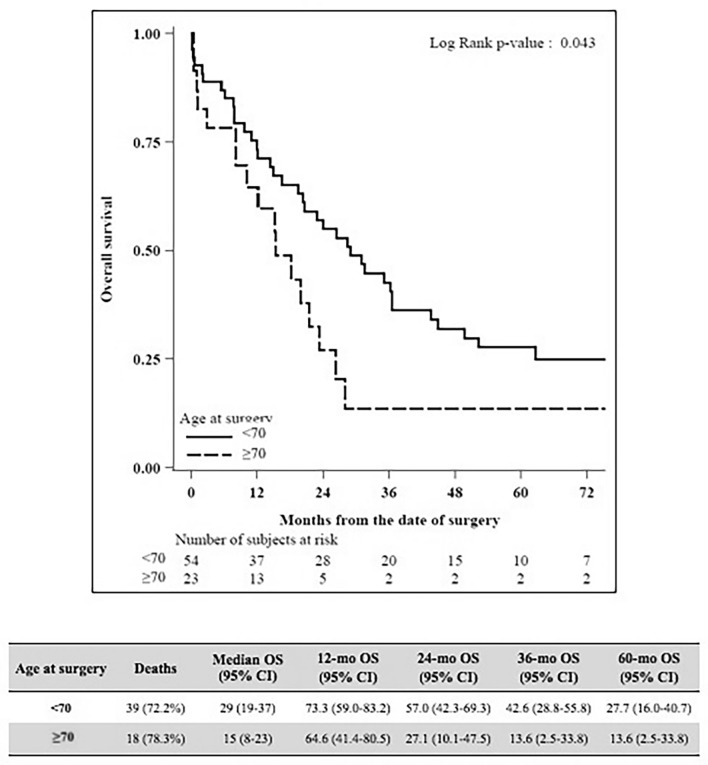
Overall survival

**Fig. 3:**
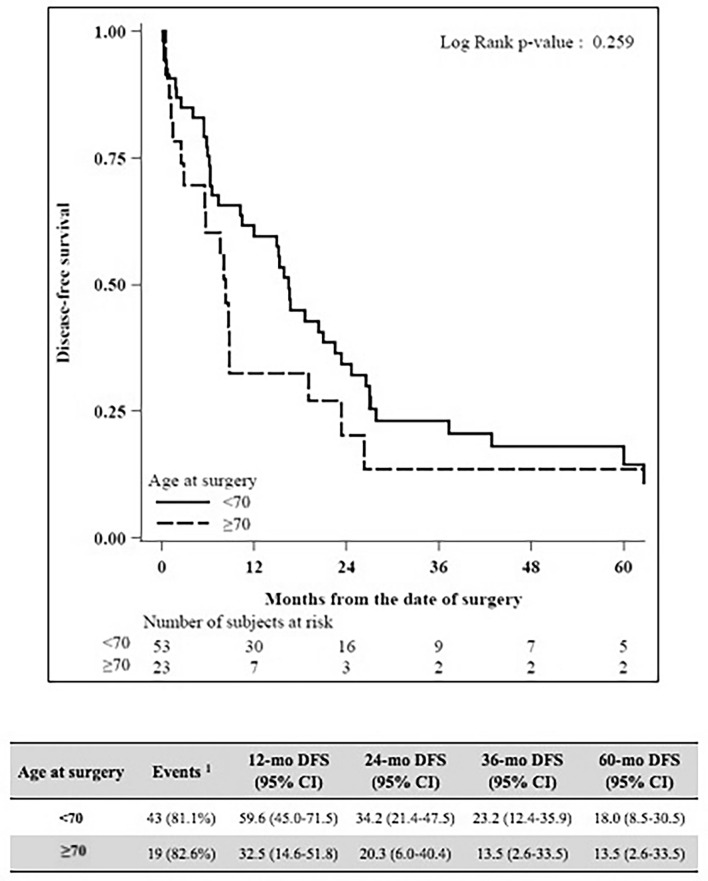
Disease-free survival

Between January 2009 and December 2019, overall 77 patients resected for PHCC were included in the study. As shown in Table 1 at the baseline, the groups were significantly different in terms of hypertension, ASA score, and Performance Status. Patients who underwent percutaneous or endoscopic biliary drainage were 25 (46.3%) in the < 70-year-old group and 11 (47,8%) in the ≥ 70-year-old group respectively (*P* = 1.00). No significant differences were found considering surgery and preoperative management (Table 2). In the < 70-year-old group, 18 (33.3%) patients had right hepatectomy, 3 (5.6%) had right trisectionectomy, 32 (59.3%) had left hepatectomy, and 1 (1.9%) had left trisectionectomy. In the ≥ 70-year-old group, 10 (43.5%) patients had right hepatectomy, 1 (4.3%) had right trisectionectomy, 15 (52.2%) had left hepatectomy, and none had left trisectionectomy (*P* = 0.88). No differences between the two groups also considering Bismuth–Corlette classification, pathology and TNM staging (Table 3). Overall R0 resection was obtained in 77.1% of cases, 74% in the < 70-year-old group and 85% in the ≥ 70-year-old group. Concerning postoperative short-term outcomes (Table 4) there are no differences between the two groups. Median LOS was 19 (5–99) for < 70-year-old group and 19 (8–90) for ≥ 70-year-old group (*P* = 0.72). The total rate of complications after surgery was 87.0% for < 70-year-old group, 73.9% ≥ 70-year-old group. Nevertheless, no differences in terms of severe complication were detected (44.4% Clavien–Dindo 3–4–5 in < 70-year-old group vs 47.8% in ≥ 70-year-old group, *P* = 0.60). The majority of severe complications were perihepatic fluid collection treated with percutaneous drainage. A biliary fistula was diagnosed in 9 (16.6%) patients in the < 70-year-old group and in 3 (12.5%) patients in the ≥ 70-year-old group. Median CCI was 30.8 (30.8 in < 70-year-old group and 29.6 in ≥ 70-year-old group, *P* = 0.85). A total of 11 patients (14.5%) died within 90 postoperative days, 6 in < 70-year-old group (11.3%), and 5 in ≥ 70 -year-old group (21.7%), *P* = 0.29. The median follow‐up for the whole sample was 20 months (IQR 8–37). The death rate was 72.2% and 78.3% among patients < 70 years old and ≥ 70 years old, respectively. The median OS was 29 (95% CI 19–37) months for the < 70-year-old group and 15 (95% CI 8–23) for the ≥ 70-year-old group. The OS was significantly higher among the < 70 years old (57.0% and 27.7% at 2 and 5 years, respectively) compared to the ≥ 70 years old (27.1% and 13.6% at 2 and 5 years, respectively, *P* = 0.043), as shown in Fig. 2. Adjusting for hypertension and Charlson comorbidity index in a multivariate Cox proportional hazard regression model, the HR for age was 1.93 (95% CI 0.84–4.44), with *P* = 0.12. Relapse occurred in 43 (81.1%) patients in the < 70-year-old group and in 19 (82.6%) patients in the ≥ 70-year-old group. DFS at 12, 24, and 36 months was, respectively, 59.6 (45.0–71.5), 34.2 (21.4–47.5), and 23.2 (12.4–35.9) for the < 70-year-old group and 32.5 (14.6–51.8), 20.3 (6.0–40.4), and 13.5 (2.6–33.5) for the ≥ 70-year-old group (*P* = 0.26), as shown in Fig. 3. Adjusting for hypertension and Charlson comorbidity index in a Cox model, the HR for age was 1.52 (95% CI 0.67–3.46), with *P* = 0.32.

**Table 1:** Baseline characteristics

Variable	Level	Overall (*N* = 77)	Age at surgery		*P* value
			< 70, (*N* = 54)	≥ 70, (*N* = 23)	
Age, median (min–max)		66 (28–85)	61 (28–69)	73 (70–85)	–
Diabetes mellitus, *N* (%)	No	70 (94.6)	50 (96.2)	20 (90.9)	0.58
	Yes	4 (5.4)	2 (3.8)	2 (9.1)	
	Missing	3	2	1	
Hypertension, *N* (%)	No	48 (64.9)	42 (80.8)	6 (27.3)	< 0.001
	Yes	26 (35.1)	10 (19.2)	16 (72.7)	
	Missing	3	2	1	
Cardiovascular comorbidity, *N* (%)	No	69 (93.2)	49 (94.2)	20 (90.9)	0.63
	Yes	5 (6.8)	3 (5.8)	2 (9.1)	
	Missing	3	2	1	
Vascular comorbidity, *N* (%)	No	70 (94.6)	50 (96.2)	20 (90.9)	0.58
	Yes	4 (5.4)	2 (3.8)	2 (9.1)	
	Missing	3	2	1	
Liver disease, *N* (%)	No	70 (94.6)	49 (94.2)	21 (95.5)	1.00
	Yes	4 (5.4)	3 (5.8)	1 (4.5)	
	Missing	3	2	1	
Respiratory disease, *N* (%)	No	65 (87.8)	47 (90.4)	18 (81.8)	0.44
	Yes	9 (12.2)	5 (9.6)	4 (18.2)	
	Missing	3	2	1	
Renal insufficiency, *N* (%)	No	71 (95.9)	51 (98.1)	20 (90.9)	0.21
	Yes	3 (4.1)	1 (1.9)	2 (9.1)	
	Missing	3	2	1	
Previous tumor, *N* (%)	No	71 (95.9)	50 (96.2)	21 (95.5)	1.00
	Yes	3 (4.1)	2 (3.8)	1 (4.5)	
	Missing	3	2	1	
Autoimmune disease, *N* (%)	No	71 (95.9)	49 (94.2)	22 (100.0)	0.55
	Yes	3 (4.1)	3 (5.8)	0 (0.0)	
	Missing	3	2	1	
Performance status (ECOG), *N* (%)	0	45 (60.8)	39 (75.0)	6 (27.3)	0.004
	1	24 (32.4)	9 (17.3)	15 (68.2)	
	2	4 (5.4)	4 (7.7)	0 (0.0)	
	3	1 (1.4)	0 (0.0)	1 (4.5)	
	Missing	3	2	1	
ASA, *N* (%)	1	28 (37.3)	25 (47.2)	3 (13.6)	0.001
	2	32 (42.7)	22 (41.5)	10 (45.5)	
	3	14 (18.7)	6 (11.3)	8 (36.4)	
	4	1 (1.3)	0 (0.0)	1 (4.5)	
	Missing	2	1	1	
Charlson Comorbidity Index, median (min–max)^a^		4 (2–8)	4 (2–7)	5 (5–8)	< 0.001

*ECOG* Eastern Cooperative Oncology Group, *ASA* American Society of Anesthesiologists

^a^3 missing values

**Table 2:** Type of surgery and preoperative management

Variable	Level	Overall (*N* = 77)	Age at surgery		*P* value
			< 70, (*N* = 54)	≥ 70, (*N* = 23)	
Preoperative portal vein embolization, *N* (%)	No	73 (94.8)	51 (94.4)	22 (95.7)	1.00
	Yes	4 (5.2)	3 (5.6)	1 (4.3)	
Preoperative dren, *N* (%)	No	41 (53.2)	29 (53.7)	12 (52.2)	1.00
	Yes	36 (46.8)	25 (46.3)	11 (47.8)	
Type of surgery, *N* (%)	Right hemihepatectomy	28 (36.4)	18 (33.3)	10 (43.5)	0.88
	Right trisectionectomy	4 (5.2)	3 (5.6)	1 (4.3)	
	Left hemihepatectomy	44 (57.1)	32 (59.3)	12 (52.2)	
	Left trisectionectomy	1 (1.3)	1 (1.9)	0 (0.0)	
Vascular resection, *N* (%)	No vascular resection	73 (94.8)	51 (94.4)	22 (95.7)	0.77
	Portal vein resection	2 (2.6)	1 (1.9)	1 (4.3)	
	Hepatic artery resection	2 (2.6)	2 (3.7)	0 (0.0)

**Table 3:** Pathology

Variable	Level	Overall (*N* = 77)	Age at surgery		*P* value
			< 70, (*N* = 54)	≥ 70, (*N* = 23)	
Grading, *N* (%)	G1	11 (15.5)	8 (15.7)	3 (15.0)	0.20
	G2	46 (64.8)	30 (58.8)	16 (80.0)	
	G3	14 (19.7)	13 (25.5)	1 (5.0)	
	Missing	6	3	3	
Resection margins, *N* (%)	R0	54 (77.1)	37 (74.0)	17 (85.0)	0.29
	R1	15 (21.4)	12 (24.0)	3 (15.0)	
	R2	1 (1.4)	1 (2.0)	0 (0.0)	
	Missing	7	4	3	
T, *N* (%)	T1-T2	46 (66.7)	31 (63.3)	15 (75.0)	0.41
	T3-T4	23 (33.3)	18 (36.7)	5 (25.0)	
	Missing	8	5	3	
N, *N* (%)	N0	43 (64.2)	27 (57.4)	16 (80.0)	0.099
	N1	24 (35.8)	20 (42.6)	4 (20.0)	
	Missing	10	7	3	
Bismuth–Corlette classification, *N* (%)	II	8 (10.4)	7 (13.0)	1 (4.3)	0.43
	IIIA	27 (35.1)	16 (29.6)	11 (47.8)	
	IIIB	38 (49.4)	28 (51.9)	10 (43.5)	
	IV	4 (5.2)	3 (5.6)	1 (4.3)

**Table 4:** Outcomes

Variable	Level	Overall (*N* = 77)	Age at surgery		*P* value of univariate analysis	*P* value of adjusted analysis^a^
			< 70, (*N* = 54)	≥ 70, (*N* = 23)		
Hospital stay, median (min–max)^b^		19 (5–99)	19 (5–99)	19 (8–90)	0.92	0.20
Clavien–Dindo, *N* (%)	0	13 (16.9)	7 (13.0)	6 (26.1)	–	–
	1	6 (7.8)	5 (9.3)	1 (4.3)		
	2	23 (29.9)	18 (33.3)	5 (21.7)		
	3a	15 (19.5)	10 (18.5)	5 (21.7)		
	3b	5 (6.5)	5 (9.3)	0 (0.0)		
	4a	5 (6.5)	4 (7.4)	1 (4.3)		
	4b	1 (1.3)	1 (1.9)	0 (0.0)		
	5	9 (11.7)	4 (7.4)	5 (21.7)		
Clavien–Dindo, *N* (%)	0	13 (16.9)	7 (13.0)	6 (26.1)	0.73	0.40
	1–2	29 (37.7)	23 (42.6)	6 (26.1)		
	3–4–5	35 (45.5)	24 (44.4)	11 (47.8)		
CCI, median (min–max)		30.8 (0–100)	30.8 (0–100)	29.6 (0–100)	0.54	0.38
Death at 90 days post-op, *N* (%)	No	65 (85.5)	47 (88.7)	18 (78.3)	0.24	0.59
	Yes	11 (14.5)	6 (11.3)	5 (21.7)		
	Missing	1	1	0		

*CCI* Comprehensive Complication Index

^a^Performing multivariate models, the effect of age at surgery was adjusted for the baseline characteristics that significantly differed between the two age groups (*P* < 0.05) at univariate analysis: hypertension and Charlson Comorbidity Index. Also PS ECOG and ASA were associated with age, but they were highly correlated with Charlson, so we excluded them from multivariate models to avoid multicollinearity)

^b^3 missing values

## Discussion

The feasibility of major liver surgery in elderly patients is discussed intensively. Social demographic trends to develop an older society. Given the increasing aged surgical population, it is not surprising that the number of elderly patients undergoing hepatic resection for treatment of liver cancer is progressively increasing. Tufo and colleagues [15] showed that the number of patients who underwent liver resection older than 70 years raised from 6% of 1990 to > 25% in 2007. Although several studies have examined the feasibility of hepatic resection for colorectal liver metastasis (CRLM), intrahepatic cholangiocarcinoma (ICC), and hepatocarcinoma (HCC) among elderly patients [8–10, 15–19], the results were disparate. Only one previous study showed that resection of PHCC under careful patient selection can be performed with low mortality irrespective of age and offers a better chance of long-term survival even in octogenarians [20]. Caution, however, is needed for interpretation of data, because nearly all previous studies involved patients who underwent simple hepatectomy without bilioenteric anastomosis. In this study, we evaluate the effect of age on postoperative outcomes among patients undergoing liver surgery for PHCC. In particular, we demonstrated that there were no differences in perioperative outcomes including postoperative complications, 90-day mortality, and length of stay (LOS) between patients younger (< 70 years old) and elderly (≥ 70 years old). Usually, elderly patients may have more comorbidities and a long hospital stay can increase complications rate and costs: in light of this, a crucial role is assigned to patients’ preoperative assessment. Cost control is nowadays a very important aspect of many health systems, which raises the question what proportion of health care resources should be allocated to the older population [19]. In the current aging society, surgeons are increasingly treating more elderly patients with cancer. Elderly patients with PHCC should not be precluded from appropriate resection of PHCC solely due to age. Although there are several scoring systems to predict postoperative mortality and morbidity, there is no evidence to indicate whether these systems are applicable for PHCC [20]. Frailty is associated with a worse survival outcome in patients with various malignancies. However, it is not a risk factor for survival in biliary tract cancer with good ECOG performance score [21]. About oncological outcomes, no difference was found for the disease-free survival (DFS), while a little difference was found between the two groups for the overall survival (OS). This finding is not unexpected, since the likelihood of death for any reason inherently increases with age progression. In the literature, Akashi and colleagues [20] reached same results about perihilar cholangiocarcinoma but evaluating an older population. That confirms that surgery does not impact in life expectancy of elderly patients. For Vitale et al. [17], both OS and DFS were comparable among elderly and non-elderly patients. In contrast, tumor characteristics were more predictive of worse survival. The current study has several limitations. This is a retrospective monocentric analysis, and the study cohort is inevitably small. Therefore, no definitive conclusions can be drawn due to the limited numbers in the two groups considered. In this context, a multicentric study would raise the number of included patients and the power of statistical testing. Moreover, the scarcity of data in the literature makes it difficult to compare our results.

## Conclusions

In the current aging society, surgeons are increasingly treating more elderly patients with cancer. Our results suggest that, despite an inferior OS, ≥ 70-year-old patients with PHCC can still be eligible for major liver resection with acceptable complication rates and perioperative results, and should not be precluded a priori from a radical treatment because only of advanced age. Other elements should be investigated to better identify elderly patients at risk of complications after major hepatic resection. Further studies evaluating a wider number of patients are needed to better define these concurrent risk factors.
